# Dehydrosqualene Desaturase as a Novel Target for Anti-Virulence Therapy against *Staphylococcus aureus*

**DOI:** 10.1128/mBio.01224-17

**Published:** 2017-09-05

**Authors:** Peng Gao, Julian Davies, Richard Yi Tsun Kao

**Affiliations:** a Department of Microbiology, Li Ka Shing Faculty of Medicine, the University of Hong Kong, Hong Kong; b Research Centre of Infection and Immunology, Li Ka Shing Faculty of Medicine, the University of Hong Kong, Hong Kong; c State Key Laboratory for Emerging Infectious Disease, the University of Hong Kong, Hong Kong; d Department of Microbiology and Immunology, the University of British Columbia, Vancouver, BC, Canada; New York University School of Medicine

**Keywords:** MRSA, anti-virulence, bacterial infection, staphyloxanthin

## Abstract

*Staphylococcus aureus*, especially methicillin-resistant *S. aureus* (MRSA), is a life-threatening pathogen in hospital- and community-acquired infections. The golden-colored carotenoid pigment of *S. aureus*, staphyloxanthin, contributes to the resistance to reactive oxygen species (ROS) and host neutrophil-based killing. Here, we describe a novel inhibitor (NP16) of *S. aureus* pigment production that reduces the survival of *S. aureus* under oxidative stress conditions. Carotenoid components analysis, enzyme inhibition, and *crtN* mutational studies indicated that the molecular target of NP16 is dehydrosqualene desaturase (CrtN). *S. aureus* treated with NP16 showed increased susceptibility to human neutrophil killing and to innate immune clearance in a mouse infection model. Our study validates CrtN as a novel druggable target in *S. aureus* and presents a potent and effective lead compound for the development of virulence factor-based therapy against *S. aureus*.

## IMPORTANCE

*S. aureus* staphyloxanthin contributes substantially to pathogenesis by interfering with host immune clearance mechanisms, but it has little impact on *ex vivo* survival of the bacterium. Agents blocking staphyloxanthin production may discourage the establishment and maintenance of bacterial infection without exerting selective pressure for antimicrobial resistance. Our newly discovered CrtN inhibitor, NP16, may offer an effective strategy for combating *S. aureus* infections.

## INTRODUCTION

Staphyloxanthin has proven to be an important factor in promoting bacterial invasion (1). Five genes, *crtOPQMN*, located in an operon are responsible for the biosynthesis of the pigment. The transcription of the operon is driven by a σ^B^-dependent promoter upstream of *crtO* and ends with a terminator downstream of *crtN* (2).

**FIG 1:**
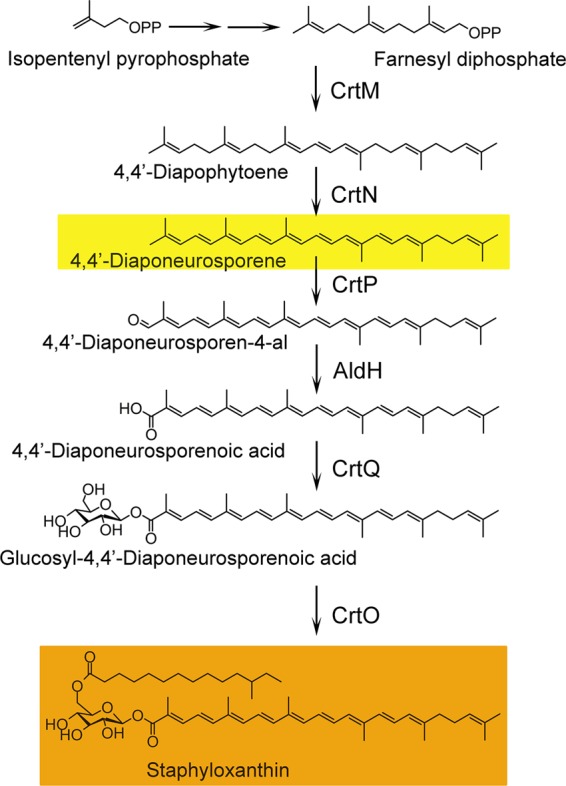
Biosynthesis pathway of staphyloxanthin. First, in staphyloxanthin biosynthesis, two molecules of farnesyl diphosphate are condensed head-to-head to form dehydrosqualene (4,4′-diapophytoene); this reaction is catalyzed by the dehydrosqualene synthase CrtM. Second, dehydrosqualene is dehydrogenated by the dehydrosqualene desaturase CrtN to form the yellow intermediate 4,4′-diaponeurosporene. Third, oxidation of the terminal methyl group of 4,4′-diaponeurosporene is catalyzed by a mixed-function oxidase, CrtP, to form 4,4′-diaponeurosporenic acid. Then, glycosyl 4,4′-diaponeurosporenoate is formed by esterification of glucose at the C-1″ position of 4,4′-diaponeurosporenic acid via CrtQ, a glycosyltransferase. Finally, glucose at the C-6″ position is esterified with the carboxyl group of 12-methyltetradecanoic acid by the acyltransferase CrtO to yield staphyloxanthin (2).

The pigments that endow *S. aureus* with a golden color (Fig. 1) also make it resistant to attack from reactive oxygen species (ROS) and neutrophils (3). Pigmented bacteria have increased resistance to the host’s immune defenses (4).

In a mouse subcutaneous model of infection, animals infected with a wild-type strain of *S. aureus* had higher bacterial loads and larger visible lesions than those infected with nonpigmented bacteria (4). The reduced virulence of bacterial strains with defective carotenoid synthesis was also shown in a mouse systemic *S. aureus* infection model (3). *In vitro* and *in vivo* data suggest that blocking pigment synthesis may reduce pathogenicity.

Dehydrosqualene synthase (CrtM), which catalyzes the first step of the biosynthetic pathway, was shown to be a target for anti-infective therapy, based on virulence factor neutralization. A drug candidate already tested in humans in the context of cholesterol-lowering therapy provides a good lead, based on its structural similarity to human squalene synthase (SQS) (3). Because of common structural features, agents selective for *S. aureus* CrtM and human SQS will have similar side effects. Diphenylamine was found to be an inhibitor of 4,4-diapophytoene desaturase (CrtN) at a high-micromolar level (5). Another potential inhibitor of CrtN, naftifine, is an FDA-approved antifungal compound shown to reduce bacterial loads in mice in different models (6). Following an established screening method for finding agents that reduce *S. aureus* pigmentation (7), we identified a compound, which we termed NP16, that blocks pigment production in *S. aureus* by targeting the 4,4-diapophytoene desaturase, a novel target proposed for anti-virulence treatments in *S. aureus*.

## RESULTS

### Compound NP16 reduces pigment production.

**FIG 2:**
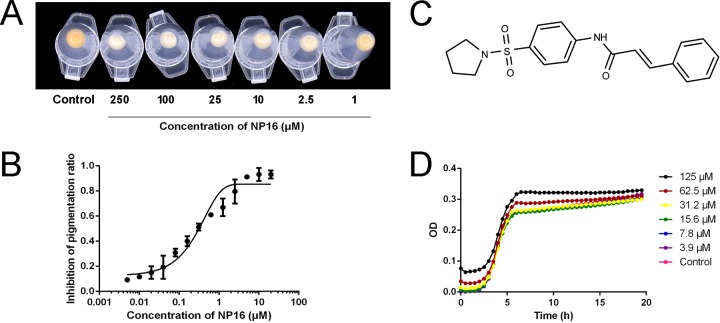
*In vitro* pigment inhibition by compound NP16. (A) Inhibition of wild-type (WT) *S. aureus* pigmentation in the presence of increasing concentrations of NP16. (B) Pigment inhibition by NP16; the IC_50_ for pigment formation is ∼300 nM. (C) The chemical structure of compound NP16. (D) Growth curve of *S. aureus* COL in the presence of different concentrations of NP16. All data represent mean values ± SD.

Compound NP16 (structure shown in Fig. 2C) has potent activity against *S. aureus* pigment formation *in vitro*, as shown in Fig. 2A, with 50% inhibitory concentration (IC_50_) 300 nM (Fig. 2B). In the biosynthesis of staphyloxanthin, the product of CrtN, 4,4′-diaponeurosporene, is a yellowish compound, while products prior to CrtM catalysis in this pathway are colorless (Fig. 1). Thus, NP16 treatment might target CrtM or CrtN or other regulators that affect the expression of the *crtOPQMN* cluster, such as *sigB* or *ispA* (8). To test whether regulators are involved, RNA samples were extracted from NP16-treated and untreated cultures, and quantitative PCR was conducted to compare *crtM* and *crtN* expression levels. No differences were observed among the tested samples. NP16 did not inhibit the growth of COL (Fig. 2D) with MIC greater than 500 μM (Fig. 2D).

Taken together, these results indicate that NP16 is an inhibitor of CrtM or CrtN.

### Inhibition of CrtN by NP16.

**FIG 3:**
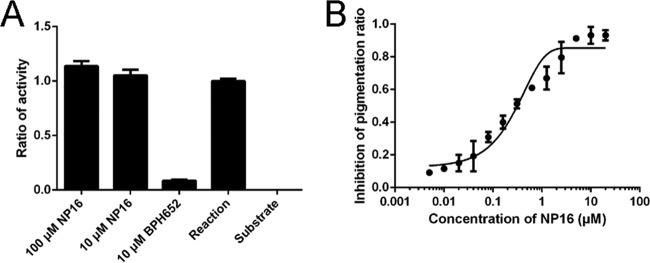
Effects of compound NP16 on CrtM and CrtN enzyme activity. (A) The inhibition of CrtM with NP16 and inhibitor BPH-652; the reaction mixture or the reaction mixture without enzyme showed activity ratios of 1 and 0, respectively. (B) An assay of CrtN enzyme activity was conducted with different concentrations of NP16, which were monitored by LC-MS. All data represent mean values ± SD.

To exclude the possibility that NP16 is a CrtM inhibitor, we purified CrtM protein (see Fig. S1 in the supplemental material) and tested the activity of CrtM to condense two farnesyl pyrophosphate (FPP) molecules. Consistent with published data (9), the CrtM inhibitor BPH652 showed nearly 100% inhibition at 10 μM, while compound NP16 showed no inhibition, even at 100 μM (Fig. 3A).

10.1128/mBio.01224-17.1 FIG S1  Purification of CrtM, determined by SDS-PAGE analysis. Lane 1, culture medium; lane 2, centrifuged bacteria; lane 3, supernatant after sonication; lane 4, pellet collected after sonication; lane 5, flowthrough after passing through the His column; lane 6, sample without IPTG induction; lane 7, sample with IPTG induction; lane 8, molecular weight ladder; lanes 9 to 15, different elution fractions. Download FIG S1, JPG file, 0.1 MB. Copyright © 2017 Gao et al. 2017 Gao et al. This content is distributed under the terms of the Creative Commons Attribution 4.0 International license.

Overexpression of N-terminal His-tagged CrtN with pQE30N (5) in *Escherichia coli* produced no soluble recombinant proteins. We constructed plasmid pET28b-*crtN* (Fig. S2A) and transformed it into the BL21(DE3) and Rosetta(DE3) strains, which showed that although CrtN production was induced with isopropyl-β-d-thiogalactopyranoside (IPTG), no soluble CrtN was detected (Fig. S2C).

10.1128/mBio.01224-17.2 FIG S2  Purification of CrtN. (A) Map of plasmid pET28b-*crtN*. (B) Map of plasmid pRhisMBP-*crtN*. (C) Purification of CrtN from pET28b-*crtN*. Lane 1, pellet collected after sonication; lane 2, supernatant after sonication. Bacteria were induced with IPTG at 16°C for 12 h., (D) SDS-PAGE results for the purification of CrtN. Lane 1, centrifuged bacteria; lane 2, pellet collected after sonication; lane 3, supernatant after sonication; lane 4, flowthrough after passing through the His column; lanes 5 to 8, different fractions after washing with less than 100 mM imidazole; lane 9, eluted CrtN; lane 10, sample after TEV treatment; lane 11, elution from His column; lane 12, flowthrough from His column. Download FIG S2, JPG file, 0.2 MB. Copyright © 2017 Gao et al. 2017 Gao et al. This content is distributed under the terms of the Creative Commons Attribution 4.0 International license.

Thus, a His-maltose binding protein (MBP) tag was introduced to increase the solubility of CrtN along with plasmid pRhisMBP-*crtN* (Fig. S2B). After treatment with tobacco etch virus (TEV) protease and removal of the MBP tag, CrtN was purified and showed a molecular mass of approximately 55 kDa (Fig. S2D).

The substrate 4,4′-diapophytoene was extracted from the carotenoid fraction from strain COL-Δ*crtN* (Fig. S3). To test CrtN activity, 4,4′-diapophytoene-containing phosphatidylcholine liposomes were made and supplemented with 15 µg of purified protein. This reaction was not active unless supplemented with FAD, as confirmed by liquid chromatography-mass spectrometry (LC-MS) analysis of the peak area of substrate.

10.1128/mBio.01224-17.3 FIG S3  Purified 4,4′-diapophytoene and LC-MS analysis results. (A) HPLC analysis results of purified 4,4′-diapophytoene. (B) MS results; the peak was 4,4′-diapophytoene. Download FIG S3, JPG file, 0.1 MB. Copyright © 2017 Gao et al. 2017 Gao et al. This content is distributed under the terms of the Creative Commons Attribution 4.0 International license.

For inhibition analysis, different concentrations of NP16 were added to the assay mixture to determine the IC_50_, and the reaction was monitored via absorbance at 286 nm. The calculated IC_50_ was 1.341 ± 0.21 μM (mean ± standard deviation [SD]) (Fig. 3B).

### NP16 treatment leads to the accumulation of 4,4′-diapophytoene.

**FIG 4:**
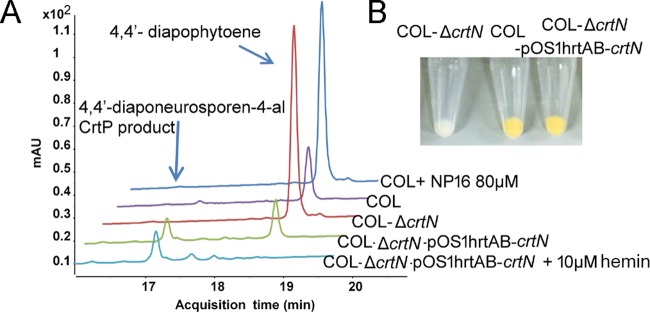
4,4′-Diapophytoene accumulation after NP16 treatment. (A) Comparison of carotenoid components among strain COL, COL-Δ*crtN*, COL-Δ*crtN-*pOS1hrtAB*-crtN*, or COL-Δ*crtN-*pOS1hrtAB*-crtN* plus 10 μM hemin (inducer), or COL treated with NP16. Plasmid pOShrtAB-crtN has a hemin-inducible promoter. (B) Comparison of the pigmentation of the wild-type COL strain, *crtN* deletion strain, and *crtN* complemented strain.

To examine the effect of NP16 on carotenoid production in *S. aureus*, the extracted carotenoids were analyzed by LC-MS. The colored components with retention times of around 17.2 min (monitored at 450 nm) were decreased in the NP16 treatment group (Fig. 4A). Compared with the untreated COL strain, compound NP16 addition revealed a peak at 18.76 min, with a molecular weight of 409.3536 (Fig. 4A; Fig. S3B), which was characterized as 4,4′-diapophytoene by LC-tandem MS (LC-MS/MS).

### Mutation and complementation.

As CrtM was previously shown to block the biosynthesis of carotenoids (4), another essential gene, *crtN*, which encodes dehydrosqualene desaturase, was investigated. To probe the biological activities of CrtN, we generated an isogenic *crtN* mutant in the COL strain via allelic replacement (Fig. S4A and B). The mutation resulted in loss of yellow pigment.

10.1128/mBio.01224-17.4 FIG S4  Isogenic mutation of the *crtN* gene in *S. aureus*. (A) Schematic representation of isogenic replacement of *crtN* with the *erm*(C) cassette. (B) PCR analysis of a *crtN*-deleted colony; lanes 2 and 4, mutant strain used as the template; lanes 3 and 5, wild-type strain used as the template. Lanes 2 and 3, primers RKP1073 and RKP1071; lanes 4 and 5, primers RKP1333 and RKP1091. (C) Plasmid map of pOS1hrtAB-crtN. Download FIG S4, JPG file, 0.1 MB. Copyright © 2017 Gao et al. 2017 Gao et al. This content is distributed under the terms of the Creative Commons Attribution 4.0 International license.

To complement this mutant strain, pOS1hrtAB-*crtN*, with a hemin-inducible promoter, was constructed (Fig. S4C). The complementation restored the pigment production of COL-Δ*crtN* to the wild-type COL level (Fig. 4B).

In comparison with the wild-type COL strain, COL-Δ*crtN* showed increased production of 4,4′-diapophytoene (Fig. 4A), without any production of pigmented carotenoids (Fig. 4A). Also, LC-MS analysis showed that the COL-Δ*crtN* strain had a comparable amount of 4,4′-diapophytoene as the NP16-treated wild-type COL strain, which also indicated that CrtN is the target of NP16.

Without NP16 treatment, hemin induced pigment production in the COL-pOS1hrtAB-*crtN* strain. However, in the parent strain carrying pOS1hrtAB, hemin failed to induce pigment production, confirming that induced pigment production is a result of the increased expression of *crtN* in the wild-type COL strain.

**FIG 5:**
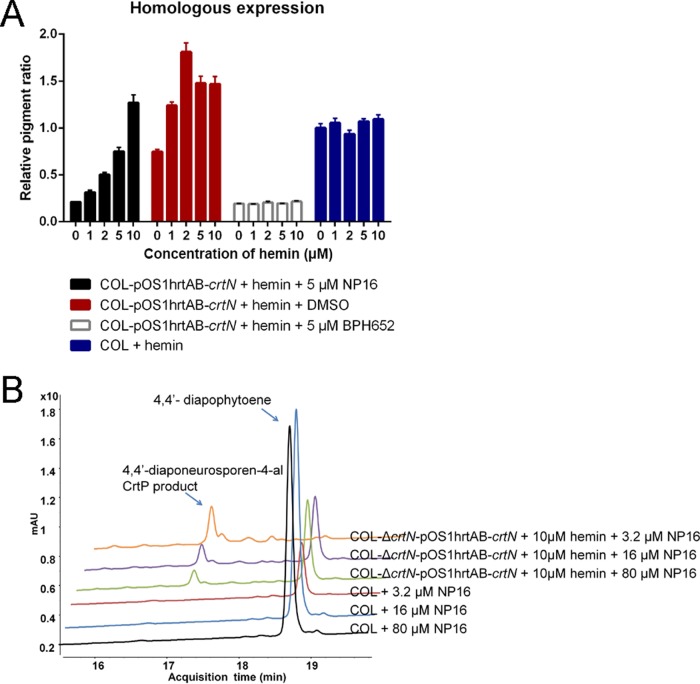
Homologous expression and intracellular inhibition of CrtN by NP16. (A) Homologous expression of *crtN* in the wild-type COL strain, treated with NP16, and with BPH652 combined with different concentrations of hemin to confirm the reduced inhibition ratio when *crtN* was overexpressed. (B) LC-MS analysis of the effects of different concentrations of NP16 on the *crtN*-overexpressing strain and the parent strain. All data represent mean values ± SD.

Compared with the dimethyl sulfoxide (DMSO) vehicle control group, compound NP16 treatment at 5 μM significantly reduced pigment production in the absence of hemin, whereas with the addition of increasing concentrations of hemin, inhibition faded away when CrtN was overproduced. In the event that high levels of CrtN are involved in other pathways, CrtM’s inhibitor, BPH652, was also included in the assay mixture, and dramatic inhibition was shown, irrespective of the hemin concentration (Fig. 5A; Fig. S5).

10.1128/mBio.01224-17.5 FIG S5  Homologous expression and intrabacterial inhibition of CrtN activity by NP16. Homologous expression of *crtN* in the wild-type COL strain, with different treatments of compound NP16, and BPH652 combined with different concentrations of hemin to verify the reduced inhibition ratio when *crtN* was overexpressed. Download FIG S5, JPG file, 0.1 MB. Copyright © 2017 Gao et al. 2017 Gao et al. This content is distributed under the terms of the Creative Commons Attribution 4.0 International license.

As the production of pigment monitored by absorbance may not directly reflect the activity of CrtN in bacterial cells, LC-MS was employed to analyze the substrate of CrtN and the product of CrtP, to investigate enzyme activity and inhibition.

Deletion of *crtN* led to the accumulation of 4,4′-diapophytoene, whereas the complementary expression of CrtN in the mutant strain, without the addition of hemin, reduced the level of 4,4′-diapophytoene back to that of the parent strain (Fig. 4A). When *crtN* expression was increased by adding 10 μM hemin, 4,4′-diapophytoene was consumed and 4,4′-diaponeurosporen-4-al, the product of CrtP with a retention time around 17.2 min, accumulated (Fig. 4A).

Thus, the proportions of 4,4′-diaponeurosporen-4-al and 4,4′-diapophytoene can be used to indicate active CrtN. Different concentrations of compound NP16 were used to test such inhibition in the wild-type COL strain and a CrtN high-expression strain. In the wild-type COL strain, 16 μM NP16 completely blocked the biosynthesis of pigment, while in the CrtN production strain, 80 μM NP16 did not block the reaction (Fig. 5B).

Taken together, these results show that compound NP16 reduced pigment production through inhibition of CrtN in bacterial cells.

### NP16 has low cytotoxicity and enhances H_2_O_2_ and neutrophil killing.

**FIG 6:**
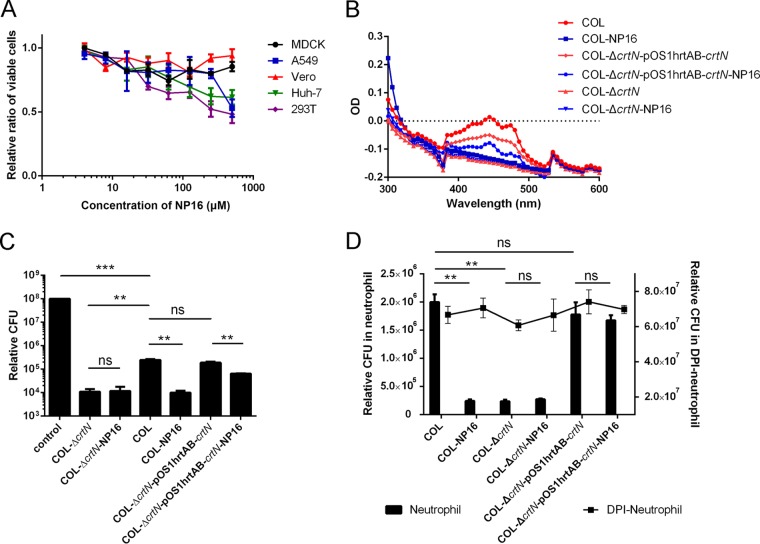
NP16 treatment leads to increased sensitivity to oxidation and neutrophil killing. (A) Cytotoxic activity of compound NP16 on different cell lines. (B) UV spectrum of carotenoids extracted from different strains, with or without NP16 treatment. (C) Increased susceptibility of the NP16-treated *S. aureus* COL strain to killing by hydrogen peroxide. (D) Increased susceptibility of the NP16-treated *S. aureus* COL strain to killing by neutrophils. All data represent mean values ± SD. ns, not significant; *, *P* < 0.05; **, *P* < 0.01; ***, *P* < 0.001; ****, *P* < 0.0001. *P* values were determined using GraphPad Prism with an unpaired parametric *t* test and Welch’s correction.

Compound NP16 had little cytotoxicity in MDCK, Vero, A549, Huh-7, or 293T cells, with 50% toxic concentrations higher than 500 µM (Fig. 6A). We found a decrease in pigment production in *S. aureus* strain COL grown in the presence of NP16 (Fig. 6B). However, for the COL-Δ*crtN*-*pcrtN* strain, the *crtN* overexpression strain, the inhibition by NP16 was decreased. In the mutant strain, with or without compound treatment, the UV spectrum patterns were similar.

Blocking *S. aureus* pigment formation led to an increase in the susceptibility of the pathogen to killing by 1.5% hydrogen peroxide exposure (Fig. 6C). Compared with the normally pigmented strain COL treated with the DMSO control, the COL-Δ*crtN* mutant and wild-type COL treated with 40 µM NP16 were killed more effectively by hydrogen peroxide by a factor of ∼16. For the nonpigmented strain COL-Δ*crtN*, the susceptibility was similar, irrespective of NP16 treatment (Fig. 6B). When *crtN* was overexpressed in COL, the ability of NP16 to increase the susceptibility of *S. aureus* to H_2_O_2_ treatment was less obvious.

Neutrophils are the immune system’s key defenders against bacterial infections; their primary function is to ingest and destroy invading pathogens. Bactericidal activity can be quantified by measuring the loss in viability of bacteria cocultured with human neutrophils (10). As a carotenoid-producing strain (Fig. 6B), COL survived significantly better than strain COL-Δ*crtN* or NP16-treated COL in human neutrophils (Fig. 6D). When neutrophils were treated with diphenyleneiodonium (DPI), a general inhibitor of flavoproteins including NADPH oxidase, which abrogates the ability of neutrophils to elicit oxidative killing of bacteria (10), both COL and NP16-treated COL cells showed similar survival rates (Fig. 6D), indicating a pivotal role of *S. aureus* carotenoid in protecting the pathogen from neutrophil-mediated oxidative killing and the specificity of NP16 in disarming this crucial protective mechanism of the invading bacterium.

### Animal studies.

**FIG 7:**
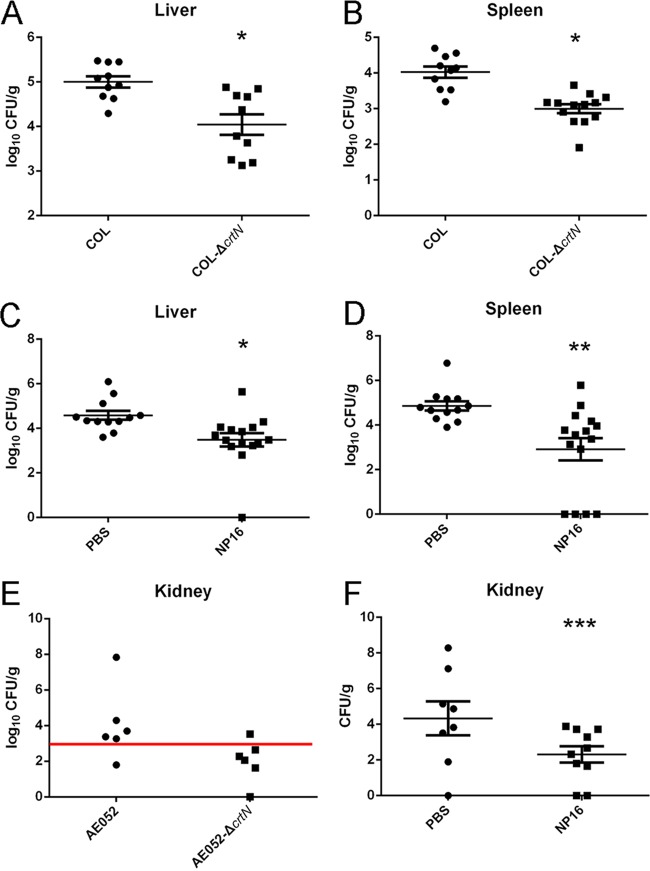
*In vivo* effect of CrtN and its inhibition by NP16. (A and B) Bacteria recovered from the spleens and livers of mice infected with the wild-type COL or COL-Δ*crtN* strains. (C and D) Bacteria recovered from the spleens and livers of mice infected with the COL strain, with or without compound NP16 treatment. (E) Bacteria recovered from the kidneys of mice infected with clinical isolate strain AE052 or strain AE052-Δ*crtN*. (F) Bacteria recovered from the kidneys of mice infected with strain AE052, with or without compound NP16 treatment. All data represent mean values ± the standard errors of the means. *, *P* < 0.05; **, *P* < 0.01; ***, *P* < 0.001. *P* values were determined using GraphPad Prism with an unpaired parametric *t* test and Welch’s correction.

Using a systemic *S. aureus* infection model, the enzyme CrtM from *S. aureus* was identified to be a target for anti-infective therapy, based on virulence factor neutralization (3). A similar model was applied to determine if *crtN* is also essential for infections in mice. The loss of staphyloxanthin reduced the invasive disease potential, as mice inoculated with the isogenic *S. aureus* mutant strain COL-Δ*crtN* showed smaller bacterial populations in the liver (*P* = 0.0018) and spleen (*P* = 0.000016) than mice inoculated with 4 × 10^8^ CFU of wild-type *S. aureus* (by intraperitoneal injection), which led to a sustained infection (Fig. 7A and B). Because the COL strain is a low-virulence strain, no bacteria were detected in the kidneys from days 1 to 3.

Another highly virulent clinical isolate, AE052, and its isogenic *S. aureus* mutant lacking the CrtN enzyme were also examined in these tests. Compared to the wild-type strain, the mutant strain in the kidney was cleared by the host after 72 h postinfection (Fig. 7E).

With the same intraperitoneal challenge used for the experiment shown in Fig. 7A, B, and E, one group of mice (*n* = 14) was treated with 0.35 mg of NP16 twice per day (days −1, 0, 1, and 2) and a second group (*n* = 12) was treated with the vehicle control. Upon sacrificing the mice at 72 h, *S. aureus* COL bacterial counts in the livers (*P* = 0.0085) and spleens (*P* = 0.0032) of mice treated with compound NP16 were significantly lower than those of the control group (Fig. 7C and D). In the case of AE052 infections, bacterial counts in the kidneys of the mice (*n* = 10 for both groups) treated with NP16 were significantly lower than those of the control group (*P* = 0.0465), with levels in 6 of 10 animals below the detection threshold, compared to undetectable levels in only 2 of 10 animals in the control group (Fig. 7F). This result indicates a 98% decrease in surviving bacteria in the treatment groups infected with COL or AE052.

## DISCUSSION

CrtM and CrtN are key enzymes in staphyloxanthin biosynthesis (11). The first step of the pathway, dehydrosqualene synthesis, is similar to an early step in human cholesterol biosynthesis (3). There is 30% sequence identity between the human SQS and the bacterial CrtM, and they share significant structural features. As a cholesterol-lowering agent and the most potent inhibitor of CrtM, BPH652 preclinical animal testing and two human clinical trials have been completed (12, 13). It provides a basis for rational drug design for use against *S. aureus* and provides proof of principle for the utility of an anti-infective drug without direct bactericidal properties that renders a pathogen susceptible to normal host innate immune clearance. The presence of the homologue of CrtM, human SQS, has discouraged the employment of CrtM as a druggable target, and a study focused on improving the specificity of BPH652 against CrtM was published recently (9). Compared with CrtM, CrtN has no homologous enzyme in the human cholesterol biosynthesis pathway, making it an attractive drug target. A recently proposed CrtN inhibitor, nafitifine, is a topically administered antifungal compound (14) that has been shown to suppress chemotaxis, chemokinesis, chemiluminescence, and superoxide anion production of polymorphonuclear leukocytes when the inhibitor is present at high concentrations (15). The effects of naftifine are not stable in different organs (from no effect to a reduced bacterial load by nearly 4 logs), and results were inconsistent with a *crtN* mutant (reduced the bacterial load from 0.2 to 2 logs at most). This indicates that CrtN should not be the primary target of naftifine (6).

ROS are employed by phagocytic cells to eliminate bacteria. They are generated by NADP (NADPH) oxidase (16). The bacterial carotenoids expressed by *S. aureus* may have a protective function against these defensive molecules (4, 17). Evidence supports that a pigment-deficient *S. aureus* strain is more sensitive to oxidants, hydrogen peroxide, and singlet oxygen *in vitro*, compared to a wild-type *S. aureus* strain (1). Using an intrabacterial inhibition assay system, we showed that the isogenic *crtN* mutant, which exhibited interrupted carotenoid synthesis, was more sensitive to purified human neutrophils. This confirmed the importance of CrtN in the intracellular survival of *S. aureus*. However, in DPI-treated neutrophils, bacterial killing appeared to be completely inhibited, illustrating that the strong effect on clearance of nonpigmented *S. aureus* should be mainly contributed by ROS in neutrophils. The compound NP16 showed a negligible effect on increasing susceptibility of the *crtN* mutant *S. aureus* strain, and the *crtN S. aureus* mutant overexpressed hydrogen peroxide in neutrophils, indicating the specific inhibition of NP16 on CrtN. A previous study reported that staphyloxanthin did not contribute to nasal colonization of *S. aureus* in mice (3). The likely reason for the latter finding is that the infection of mucosal surfaces does not cause inflammation, as the role of staphyloxanthin is to resist the host’s oxidant-based phagocytic defenses.

We believe that CrtN is a novel target for a virulence factor-based therapy against *S. aureus*. CrtN inhibitors without direct bactericidal properties render the pathogen susceptible to normal host innate immune clearance. Our approach, as well as other virulence factor-based concepts (3, 18) for highly specific antistaphylococcal therapy, also offers theoretical advantages for reducing the selection pressure that favors the emergence of drug resistance, both in the pathogen and in normal commensal microflora.

## MATERIALS AND METHODS

### Bacteria, mice, and chemical reagents.

Plasmids used in this study are listed in Table S1 and *S. aureus* strains and *E. coli* strains are listed in Table S2. BALB/c mice were purchased from Charles River Laboratories, Inc. *S. aureus* was propagated in Terrific broth (TB) or on TB agar (Life Technologies, Inc.). Unless otherwise indicated, all experiments were performed with bacteria derived from light-protected *S. aureus* 36- to 48-h stationary-phase cultures, the point at which pigmentation phenotypes were readily apparent.

10.1128/mBio.01224-17.6 TABLE S1  Plasmids used in this study. Download TABLE S1, DOCX file, 0.01 MB. Copyright © 2017 Gao et al. 2017 Gao et al. This content is distributed under the terms of the Creative Commons Attribution 4.0 International license.

10.1128/mBio.01224-17.7 TABLE S2  Strains used in this study. Download TABLE S2, DOCX file, 0.01 MB. Copyright © 2017 Gao et al. 2017 Gao et al. This content is distributed under the terms of the Creative Commons Attribution 4.0 International license.

### MIC tests.

MICs were determined by inoculating 5 × 10^4^ *S. aureus* cells in 100 μl brain heart infusion (BHI) medium in 96-well plates with a serial dilution of antibiotics. The MIC was defined as the minimum concentration resulting in a cell density less than 0.01 optical density (OD) units at 620 nm, which corresponded to no visible growth, after incubation for 18 h at 37°C.

### Cytotoxicity evaluation of NP16 in different cell lines.

The cytotoxicity of NP16 in MDCK, A549, Vero, Huh-7, and 293T cells was evaluated in an MTT (3-[4,5-dimethylthiazol-2-yl]-2,5-diphenyltetrazolium bromide) (Sigma, Hong Kong) assay according to the manufacturer’s instructions. A toxic control (1% SDS) was included to ensure that the MTT assay was effective. The highest concentration of NP16 that could be used was 500 μM, due to solubility limitations. SigmaPlot (version 11; Systat Software, Inc., San Jose, CA) was used for graph plotting. Experiments were repeated twice in triplicate.

### Real-time PCR.

The preparation of total RNA from *S. aureus* was performed using RNAprotect reagent (Qiagen, Germany) according to the manufacturer’s instructions. Briefly, total RNA was prepared by lysostaphin extraction using 5 × 10^8^ CFU of bacteria at each time point, followed by further purification with an RNeasy kit (Qiagen, Germany) according to the manufacturer’s instructions. The quality and quantity of total RNA were confirmed by agarose electrophoresis and UV spectrophotometry, respectively.

Contaminating chromosomal DNA was removed by DNase treatment (Life Technologies, Inc., Hong Kong). Purified *S. aureus* RNA was reverse transcribed into cDNA by using SuperScript III first-strand synthesis supermix (Life Technologies, Hong Kong) and then subjected to real-time PCR analysis using an ABI 7500 thermocycler (Life Technologies, Inc.) and Fast SYBR Green master mix (Life Technologies, Inc.). The relative quantification of *S. aureus* transcripts was determined by the ratio of expression of target transcripts relative to expression of *gyrB*. The primers (Table S3) used for real-time PCR experiments were RKP1017 and RKP1018 for *crtN* and RKP1019 and RKP1020 for *crtM*.

10.1128/mBio.01224-17.8 TABLE S3  Primers used in this study. Download TABLE S3, DOCX file, 0.01 MB. Copyright © 2017 Gao et al. 2017 Gao et al. This content is distributed under the terms of the Creative Commons Attribution 4.0 International license.

### Cloning of *crtM* and *crtN.*

Full-length *crtM* was amplified by PCR from *S. aureus* genomic DNA using the primers RKP875 and RKP876. The complete coding sequence of *crtM* was digested with NcoI and XhoI and cloned into the expression vector pET28b (Novagen, Madison, WI).

*crtN* was amplified by PCR from *S. aureus* genomic DNA using primers RKP1325 and RKP1326. The *crtN* gene was digested with BamHI and XhoI and cloned into the expression vector phisMBP (19).

### CrtM expression, purification, and inhibition.

*E. coli* Rosetta(DE3) cells were used to overexpress histidine-tagged CrtM. An overnight culture was diluted 1% into LB medium containing 50 μg/ml kanamycin. Induction was carried out with 1 mM IPTG for 12 h at 20°C, when the cell culture reached an OD of 0.6 at 600 nm. The cell extract was centrifuged and loaded onto a Ni-nitrilotriacetic acid (NTA) column, and CrtM was eluted by using a 75-ml linear gradient of 0 to 0.4 M imidazole in 50 mM Tris-HCl buffer, pH 7.4.

The CrtM activity assay was carried out in reaction buffer (50 mM Tris-HCl, 1 mM MgCl_2_, 450 μM FPP; pH 7.4). The compounds investigated were preincubated with 2 μg of CrtM for 30 min at 20°C.

All reaction mixtures were incubated at 37°C for 40 min, reactions were terminated by the addition of 10 μl of β-mercaptoethanol, and the reaction mixtures were then added to 40 μl of coloration reagent prepared with 2.5% ammonium molybdate in 2.5 M H_2_SO_4_. Absorbance (the OD at 595 nm) was recorded to calculate the relative activity of CrtM.

The IC_50_ values were obtained by fitting the inhibition data to a normal dose-response curve using SigmaPlot (version 11; Systat Software, Inc., San Jose, CA).

### CrtN expression and purification.

CrtN with a histidine-MBP tag was overexpressed in *E. coli* Rosetta(DE3) cells. A 10-ml overnight culture was transferred into 1 liter of LB medium supplemented with 100 μg/ml ampicillin. Induction was carried out with 1 mM IPTG for 12 h at 16°C at an OD of 0.6 at 600 nm. The cell lysate was loaded onto a Ni-NTA column, and CrtN was eluted using a 75-ml linear gradient of 0 to 0.4 M imidazole in 50 mM sodium-phosphate buffer with 400 mM sodium chloride at pH 6.6. The collected fractions were analyzed by SDS-PAGE to confirm the peak for MBP-CrtN. The target peak fractions were concentrated, and the buffer was exchanged to loading buffer without imidazole and using a PD-10 column (GE Healthcare). The collected solution was treated with TEV protease at 4°C overnight. The protein sample was applied to a maltose column, and the flowthrough was collected as native CrtN protein.

### Isolation of carotenoids.

The substrate (4,4′-diapophytoene) and product (4,4′-diaponeurosporene) were extracted from strains COL-Δ*crtN* and COL-Δ*crtOP*. Carotenoids were extracted from cell pellets using 300 ml of methanol per liter of cultured bacterial pellet until all visible pigments were removed. After centrifugation (4°C, 8,000 × *g*), colored supernatants were pooled and concentrated to 50 ml by using an EZ-2 Plus centrifugal evaporator (Genevac Inc., Gardiner, NY). A sample was mixed with 100 ml of ethanol acetate and 200 ml of NaCl (2.5 M). The extract sample in the upper organic phase was collected, washed with same volume of distilled water, and dried using the EZ-2 Plus evaporator. Dried samples were ready for silica gel isolation or were stored at −70°C prior to analysis.

### Generation of carotenoid-deficient *S. aureus* mutants Δ*crtN* and Δ*crtOP.*

Allelic replacement of the *S. aureus crtN* gene with an erythromycin resistance gene [*erm*(C)] cassette was performed using PCR-based methods, as previously described (20). PCR was used to amplify an ∼1,000-bp upstream fragment of *crtN* by using the primers RKP1067 and RKP1068, along with an ∼1,000-bp fragment immediately downstream of *crtN* by using the primers RKP1069 and RKP1070. The plasmid pCL52.2K-ErmC was digested with PstI and HindIII and ligated with the upstream PCR product, and then the same process was repeated for the downstream PCR product, using BamHI and EcoRI. The generated plasmid was designated pCL52.2K-*crtN*, and the gene was confirmed by sequencing (Beijing Genomics Institute [BGI], Hong Kong). This vector was transformed initially into the permissive *S. aureus* strain RN4220 and then into *S. aureus* strains COL and AE052 by electroporation. Transformants were grown at 30°C and then shifted to the nonpermissive temperature for plasmid replication (42°C). The shift was repeated three times, and colonies grown on erythromycin-containing plates rather than erythromycin plus kanamycin plates were identified as candidate mutants. Allelic replacement of the *crtN* allele was confirmed by pigment phenotype and PCRs documenting the targeted insertion of *erm*(C) and the absence of *crtN* in chromosomal DNA isolated from the final mutant, Δ*crtN*.

For the deletion of *crtOP*, pKOR1-mediated gene replacement was performed as described previously (21). Briefly, sequences upstream and downstream of *crtOP* were amplified using primers RKP1530/RKP1531 and RKP1532/RKP1533, respectively. Another PCR using primers RKP1530 and RKP1532 was conducted to amplify the deletion fragment. This fragment was then cloned into pKOR1. Following cloning and *ccdB* selection in *E. coli*, the constructed plasmid was then introduced into strain COL by electroporation using a GenePulser Xcell apparatus (Bio-Rad Laboratories, Inc., Hercules, CA). Gene replacement colonies were selected according to previously published procedures (21). To check for successful introduction of the mutations, the resulting mutants were checked by PCR using primers RKP1552 and RKP1553 and for the target sequences via sequencing. Colonies carrying the expected mutation that survived were designated mutants.

### Extraction of 4,4′-diapophytoene and 4,4′-diaponeurosporene.

To purify 4,4′-diapophytoene, carotenoids collected from strain COL-Δ*crtN* were dissolved in hexane and separated on a silica gel column (2.0 by 30 cm) with pure hexane. To purify 4,4′-diaponeurosporene, carotenoids collected from the COL-Δ*crtOP* mutant were dissolved in hexane and separated on a silica gel column (2.0 by 30 cm) with pure hexane, followed by increasing the concentration of ethyl acetate to 2% in hexane. The flowthrough was collected every 10 ml.

The fragments were monitored by thin-layer chromatography (TLC) with a 4:1 petroleum ether:diethyl ether solvent system, and the spots were visualized using 10% phosphomolybdic acid (PMA) in ethanol.

### LC-MS analysis.

A 10-μl aliquot of the crude extract or the collected fraction was applied to a YMC-Pack ODS-A column (4.6 by 150 mm, 5.0 μm; YMC America, Inc., Allentown, PA) and eluted under isocratic conditions with a solvent system (isopropanol from 50% in water with 0.1% trifluoroacetic acid for 5 min, then a gradient from 50% to 95% in 25 min, and then kept at 95% for 5 min) at a flow rate of 1 ml/min using an Agilent 1200 HPLC system equipped with a photodiode array detector. For structural elucidation, carotenoids were identified using a combination of high-performance LC (HPLC) retention times, UV-visible absorption spectra, and mass fragmentation spectra. Mass fragmentation spectra were monitored using both negative and positive ion modes in a mass range of *m/z* 200 to 1,000 on the Varian 1200L LC-MS system equipped with an atmospheric pressure chemical ionization interface.

### Preparation of 4,4′-diapophytoene liposomes and CrtN reaction mixtures.

Ten milligrams of phosphatidylcholine (Sigma-Aldrich) was dissolved in 1 ml of CHCl_3_. Then, 100 nmol of 4,4′-diapophytoene was added to the mixture. The lipid-phytoene mixture was dried, dissolved with 2 ml of buffer I (50 mM Tris-HCl [pH 8.0], 100 mM NaCl) on ice for 30 min, and then sonicated on ice for 5 min.

For the enzyme activity assay, 10 µg of purified CrtN was incubated with 100 µl of 4,4′-diapophytoene liposomes (containing 5 nmol of 4,4′-diapophytoene), 150 µM flavenine adenine dinucleotide, and buffer II (20 mM phosphate buffer [pH 8.0], 100 mM NaCl) in a total volume of 660 µl at 37°C for 2 h (standard assay). The reaction was stopped by adding 1 vol of CHCl_3_:methaol (MeOH; 2:1 [vol/vol]). Following mixing, the sample was centrifuged at 16,000 × *g* for 10 min. The organic phase was dried for LC-MS analysis.

### Complementation.

Primers RKP1333 and RKP1191 were used to amplify the *crtN* gene from the chromosome of the wild-type *S. aureus* COL strain. The fragment was cloned into the vector pOS1hrtAB (22), and the recombinant plasmid (pOS1hrtAB-*crtN*) was transformed via electroporation into the *S. aureus* COL-Δ*crtN* mutant. It included a hemin-inducible promoter hrtAB, and 10 µM hemin was sufficient for inducing expression of the target gene.

### Homologous expression and *ex vivo* enzyme inhibition assay.

The wild-type COL strain and the COL strain transformed with pOS1hrtAB-*crtN* were cultured with different concentrations of hemin, ranging from 0 to 10 µM. Compounds NP16 and BPH652 were added to different cultures at a concentration of 10 µM. Five-milliliter cultures in 50-ml Falcon tubes were incubated at 37°C, with shaking at 250 rpm, for 24 h. Bacteria were collected for the extraction of carotenoids. The extracted carotenoids were analyzed with a plate reader at an OD of 450. The inhibition ratio of compound NP16 was compared with a DMSO control and BPH652. The wild-type COL strain with hemin added was used as a control.

The wild-type COL and COL-Δ*crtN*-pOS1hrtAB-*crtN* strains were also cultured in TB medium with appropriate antibiotics. Hemin was added to 10 µM to induce *crtN* expression in the complemented strain, and different concentrations of compound NP16, ranging from 80 µM to 0.64 µM, were added to monitor the IC_50_ of the carotenoids. Sample collection was similar to that of the homologous expression assay. After the extraction of carotenoids, LC-MS was used to analyze the inhibition by compound NP16 and to monitor *ex vivo* enzyme inhibition. The peak areas of the target component were integrated and calculated to obtain the differences in IC_50_s.

### Hydrogen peroxide susceptibility assay.

*S. aureus* was grown in BHI with or without NP16 (40 μM). After 2 days, bacteria were washed twice in phosphate-buffered saline (PBS) and diluted to a concentration of 1 × 10^7^ CFU per 100-μl reaction mixture in a 96-well plate. H_2_O_2_ in PBS was added to a 440 mM final concentration, and the plate was incubated for 1 h at 37°C with shaking. The reaction was stopped by the addition of 1,000 U/ml of exogenous catalase (Sigma-Aldrich, St. Louis, MO), and bacterial viability was assessed by plating dilutions on BHI agar plates.

### Isolation of human neutrophils.

The isolation of human neutrophils was conducted by the gradient density centrifugation method as reported by Freitas et al. (23), with Histopaque solutions 1077 and 1119 in 12-ml polypropylene centrifuge tubes. Briefly, 3 ml of Histopaque 1077 was carefully layered on top of 3 ml of Histopaque 1119 in a 12-ml polypropylene tube. Then, 6 ml of collected blood was layered on top. After centrifugation at 890 × *g* for 30 min at 20°C, the neutrophils were carefully collected using a Pasteur pipette. The neutrophils doubled in volume when we used PBS lacking Ca^2+^ and Mg^2+^. After centrifugation at 870 × *g* for 5 min at 4°C, the pellet was resuspended with a mixture of 1.25 ml of PBS (lacking Ca^2+^ and Mg^2+^) and 5.25 ml of sterile distilled water to lyse any remaining red blood cells. The tube was gently inverted for 1.30 min, and 2.2 ml of 3% NaCl was added. After another centrifugation at 870 × *g* for 5 min at 4°C, the neutrophil pellet was resuspended in PBS containing Ca^2+^ and Mg^2+^. Isolated neutrophils were kept on ice until use. Neutrophils from one volunteer were used per experiment.

### Bactericidal activity of polymorphonuclear leukocytes.

The killing of *S. aureus* by human polymorphonuclear leukocytes (PMNs) was determined as previously described (24), with some modifications. Briefly, PMNs (10^7^) treated or not with 10 µM DPI were mixed with ∼10^8^ opsonized *S. aureus* bacteria (multiplicity of infection, 10) in 24-well tissue culture plates. After centrifugation at 380 × *g* for 8 min, plates were incubated at 37°C for up to 1.5 h. PMNs were lysed with saponin (20 min on ice) and plated on BHI agar plates. The percent survival was calculated by normalization with time zero results. Statistics were performed with Student’s *t* test (GraphPad Prism).

### Murine model of intraperitoneal infection.

Eight- to 10-week-old female BALB/c mice were injected intraperitoneally (i.p.) with 4 × 10^8^ CFU of early-stationary-phase *S. aureus* COL cells. After 3 days, animals were euthanized, the livers and spleens were isolated, then homogenized in PBS, and plated to obtain viable counts. For the treatment study, mice were randomized into two groups at the start of the experiment and administered i.p. either 0.35 mg of NP16 or PBS with 5% Tween 80 as a control, twice per day, starting on day −1 to day 2 (a total of eight doses for each animal). Intraperitoneal challenge with 4 × 10^8^ CFU of early-stationary-phase *S. aureus* COL cells was performed on day 0. The mice were sacrificed on day 3 for enumeration of bacterial CFU in liver and spleen homogenates. Ten mice were used in the first experiment, and 13 were used in the second. All results were pooled for statistical analysis.

For the clinical isolate *S. aureus* strain AE052, all operations were similar to those used for the COL strain, except 10^8^ CFU of early-stationary-phase bacteria were used in the infection model, and kidneys were collected for monitoring bacterial loads. Statistics were performed using Student’s *t* test (GraphPad Prism).
