# Supplementary figures and images for: Dehydrosqualene Desaturase as a Novel Target for Anti-Virulence Therapy against Staphylococcus aureus
Source: mBio. 2017 Sep 5;8(5):e01224-17. doi: 10.1128/mBio.01224-17 (PMC5587911; doi:10.1128/mBio.01224-17)

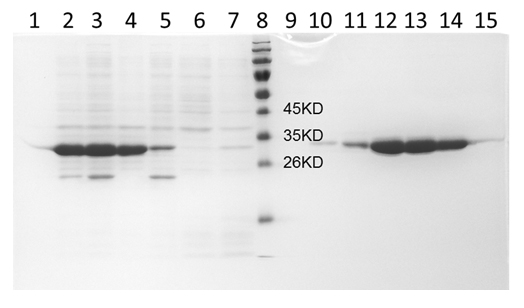

Supplement: FIG S1 [file mbo004173473sf1.jpg]

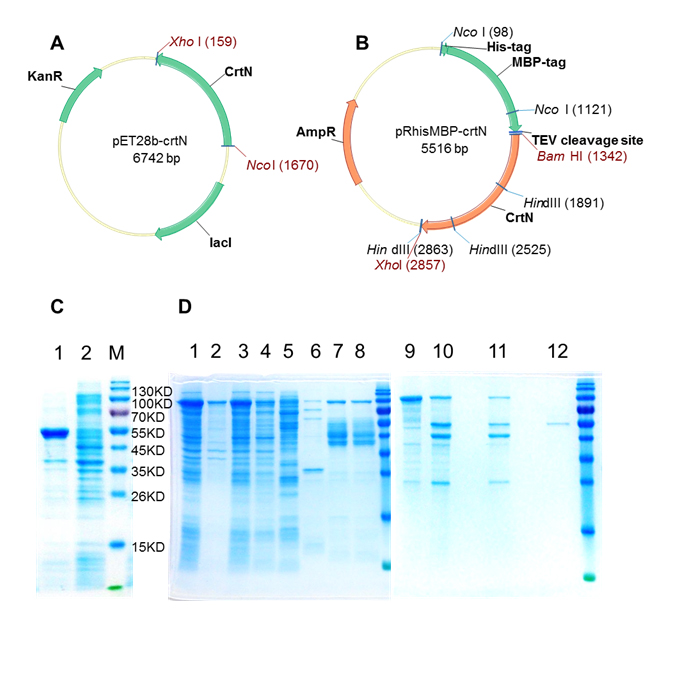

Supplement: FIG S2 [file mbo004173473sf2.jpg]

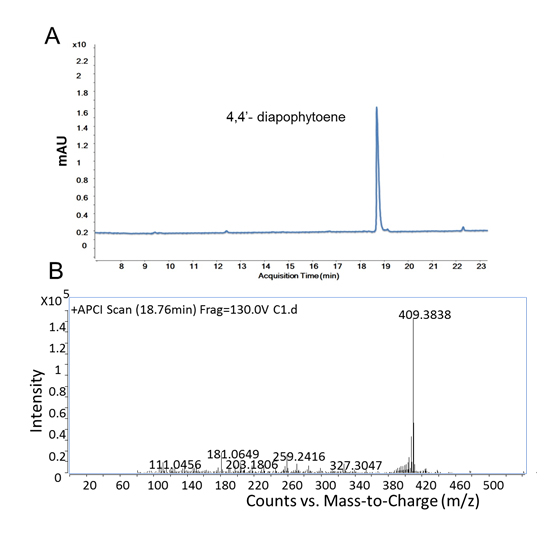

Supplement: FIG S3 [file mbo004173473sf3.jpg]

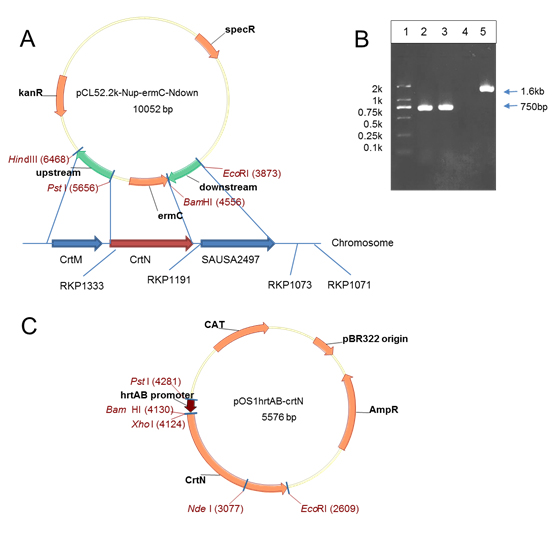

Supplement: FIG S4 [file mbo004173473sf4.jpg]

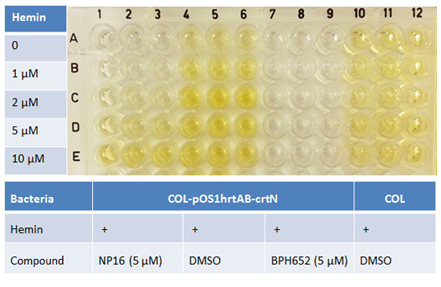

Supplement: FIG S5 [file mbo004173473sf5.jpg]
