# Supplementary material for: Dehydrosqualene Desaturase as a Novel Target for Anti-Virulence Therapy against Staphylococcus aureus
Source: mBio. 2017 Sep 5;8(5):e01224-17. doi: 10.1128/mBio.01224-17 (PMC5587911; doi:10.1128/mBio.01224-17)
Supplement: TABLE S1 [file mbo004173473st1.docx]

**Table S1. Plasmids used in this study**

| Plasmid | Description | Source |
| --- | --- | --- |
| pCL52.2K | *E*. *coli – S*. *aureus* shuttle vector for gene replacement, Spc^R^ in *E*. *coli*; Kan^R^ in *S*. *aureus* | (1) |
| pCL52.2K-*crtN* | A cassette of *crtN* upstream, ermC and *crtN* downstream inserted in pCL52.2K | This study |
| pOS1hrtAB | *E*. *coli – S*. *aureus* shuttle vector for gene expression with *hrtAB* promoter, Amp^R^ in *E*. *coli*; Chl^R^ in *S*. *aureus* | (2) |
| pOS1hrtAB-*crtN* | pOS1hrtAB plasmid with *crtN* gene | This study |
| pET28b | Plasmid for overexpression protein in *E. coli* | Lab source |
| pET28b-*crtM* | pET28b with *crtM* gene | This study |
| phisMBP | Plasmid for overexpression protein with his-tag and MBP-tag in *E. coli* | / |
| pHisMBP-*crtN* | phisMBP with *crtN* gene | This study |

**Supplementary References:**

1. Kao RY YK, Che CM, Siu FM (2011) Methionine aminopeptidase as a novel target for antibiotic therapy against Staphylococcus aureus: a proteomic approach. *Hong Kong Med J* 17(Suppl 2):29-31.

2. Benson MA, Lilo S, Nygaard T, Voyich JM, & Torres VJ (2012) Rot and SaeRS cooperate to activate expression of the staphylococcal superantigen-like exoproteins. *J Bacteriol* 194(16):4355-4365.
