# Supplementary material for: Dehydrosqualene Desaturase as a Novel Target for Anti-Virulence Therapy against Staphylococcus aureus
Source: mBio. 2017 Sep 5;8(5):e01224-17. doi: 10.1128/mBio.01224-17 (PMC5587911; doi:10.1128/mBio.01224-17)
Supplement: TABLE S2 [file mbo004173473st2.docx]

**Table S2. Strains used in this study**

| Strains | | Description | Source |
| --- | --- | --- | --- |
| *E. coli* | |  |  |
|  | Rosetta (DE3) | Host strain for gene expression | Lab source |
|  | Topo10 | Host strain for plasmids | Life technologies |
|  | Rosetta-pET*crtM* | For expressing CrtM protein | This study |
|  | BL21 (DE3)-pHisMBP*crtN* | For expressing CrtN protein | This study |
| *S. aureus* | |  |  |
|  | RN4220 | intermediate cloning host | Lab source |
|  | COL | laboratory strain | Lab source |
|  | AE052 | Clinical isolate | (1) |
|  | COL-Δ*crtN* | COL with *crtN* gene replaced with ermC cassette | This study |
|  | COL-Δ*crtN*-pOS1hrtAB-*crtN* | *crtN* mutant strains of COL complemented with *crtN* gene | This study |
|  | COL-pOS1hrtAB-*crtN* | COL homologous expressing *crtN* gene | This study |
|  | COL-pOS1hrtAB | COL with blank plasmid as control | This study |
|  | AE052-Δ*crtN* | AE052 with *crtN* gene replaced with ermC cassette | This study |

**Supplementary References:**

1. Ho PL*, et al.* (2009) Community-associated methicillin-resistant Staphylococcus aureus skin and soft tissue infections in Hong Kong. *Hong Kong Med J* 15 Suppl 9:9-11.
