# Supplementary material for: Dehydrosqualene Desaturase as a Novel Target for Anti-Virulence Therapy against Staphylococcus aureus
Source: mBio. 2017 Sep 5;8(5):e01224-17. doi: 10.1128/mBio.01224-17 (PMC5587911; doi:10.1128/mBio.01224-17)
Supplement: TABLE S3 [file mbo004173473st3.docx]

**Table S3. primers used in this study**

| No. | Primer | Enzyme site |
| --- | --- | --- |
| RKP875 | GTCGCCATGGGCATGACAATGATGGATATGAATTTTAAATATTG | Nco I |
| RKP876 | GTCGCTCGAGTATTCTATGATATTTACTATTTATTTCATG | Xho I |
| RKP1325 | GTCGCCGGATCCAAGATTGCAGTAATTGGTGCAGGTGTC | BamH I |
| RKP1326 | GTCGCTCGAGTTATACGCCCCGCTCAATATCTTTAATCA | Xho I |
| RKP1067 | CGCCAAGCTTGAAAAATACCCATATGAACATCATC | HindIII |
| RKP1068 | CGCCCTGCAGTATTCAACCACCTATATTCTATGAT | Pst I |
| RKP1069 | CGCCGGATCCGGGAGTAGTCTAAGAGAAAGATGTG | BamH I |
| RKP1070 | CGCCGAATTCATTATATTGTCTTTGATAAAAATCT | EcoR I |
| RKP083 | ACGCGTCGACTGGCTAACACACACG | Sal I |
| RKP084 | CGCGGATCCTCACAAAAAATAGGT | BamH I |
| RKP1191 | GTGTACACTCGAGTATCATAGAATATAGGTGGTTGAAT | Xho I |
| RKP1333 | CGCCGAATTCTTATACGCCCCGCTCAATATCTTTA | EcoR I |
| RKP1017 | GGTATGTATGGCATGGCTCA |  |
| RKP1018 | GGCCCGTTTGAATTTAGGAT |  |
| RKP1019 | TGATGACAGTATAGATGTTTATGG |  |
| RKP1020 | ACATGCTGAAGGGCCATCATG |  |
